# Preclinical Rodent Models for Human Bone Disease, Including a Focus on Cortical Bone

**DOI:** 10.1210/endrev/bnae004

**Published:** 2024-02-05

**Authors:** Natalie Y Y Koh, Justyna J Miszkiewicz, Mary Louise Fac, Natalie K Y Wee, Natalie A Sims

**Affiliations:** Bone Cell Biology & Disease Unit, St. Vincent's Institute of Medical Research, Fitzroy, VIC 3065, Australia; Department of Medicine at St. Vincent's Hospital, The University of Melbourne, Fitzroy, VIC 3065, Australia; School of Social Science, The University of Queensland, Brisbane, QLD 4072, Australia; Vertebrate Evolution Development and Ecology, Naturalis Biodiversity Center, 2333 CR Leiden, The Netherlands; Bone Cell Biology & Disease Unit, St. Vincent's Institute of Medical Research, Fitzroy, VIC 3065, Australia; Department of Medicine at St. Vincent's Hospital, The University of Melbourne, Fitzroy, VIC 3065, Australia; Bone Cell Biology & Disease Unit, St. Vincent's Institute of Medical Research, Fitzroy, VIC 3065, Australia; Department of Medicine at St. Vincent's Hospital, The University of Melbourne, Fitzroy, VIC 3065, Australia; Bone Cell Biology & Disease Unit, St. Vincent's Institute of Medical Research, Fitzroy, VIC 3065, Australia; Department of Medicine at St. Vincent's Hospital, The University of Melbourne, Fitzroy, VIC 3065, Australia

**Keywords:** osteoporosis, osteocytes, cortical bone, preclinical research, cortical porosity, mouse models, bone development, ageing

## Abstract

Preclinical models (typically ovariectomized rats and genetically altered mice) have underpinned much of what we know about skeletal biology. They have been pivotal for developing therapies for osteoporosis and monogenic skeletal conditions, including osteogenesis imperfecta, achondroplasia, hypophosphatasia, and craniodysplasias. Further therapeutic advances, particularly to improve cortical strength, require improved understanding and more rigorous use and reporting.

We describe here how trabecular and cortical bone structure develop, are maintained, and degenerate with aging in mice, rats, and humans, and how cortical bone structure is changed in some preclinical models of endocrine conditions (eg, postmenopausal osteoporosis, chronic kidney disease, hyperparathyroidism, diabetes). We provide examples of preclinical models used to identify and test current therapies for osteoporosis, and discuss common concerns raised when comparing rodent preclinical models to the human skeleton. We focus especially on cortical bone, because it differs between small and larger mammals in its organizational structure. We discuss mechanisms common to mouse and human controlling cortical bone strength and structure, including recent examples revealing genetic contributors to cortical porosity and osteocyte network configurations during growth, maturity, and aging. We conclude with guidelines for clear reporting on mouse models with a goal for better consistency in the use and interpretation of these models.

## Graphical Abstract

**Graphical Abstract:**
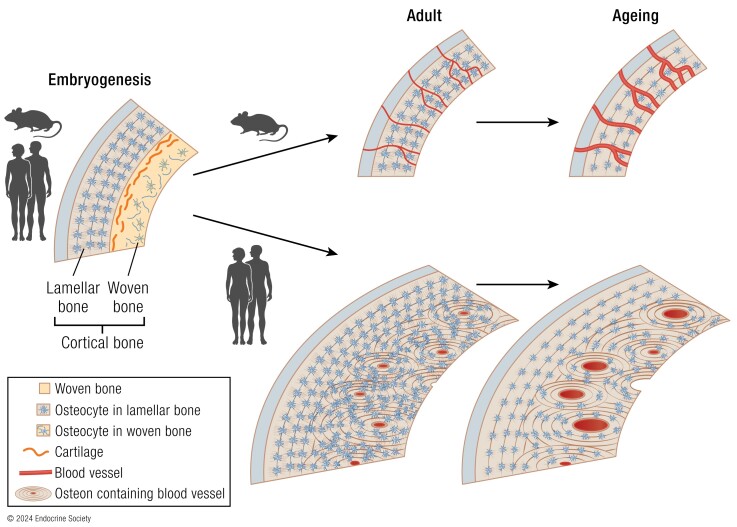

**Essential Points**

- Rodent models have been essential in the development of all current therapies for osteoporosis
- The cortical structure of small mammals differs from that of large mammals, including humans, because they have smaller cortical bone and they do not have the lifespan to develop osteonal structures
- Development and genetic control of cortical and trabecular structures are conserved between small and large mammals
- Intracortical remodeling occurs in rats and mice, even though they do not typically form osteons
- Rats and mice, like humans, exhibit increased cortical porosity and reduced connection of the osteocyte network with aging

## Background: Human Bone Diseases and Remaining Issues for Better Treatment

Determinants of bone strength, and therapies for osteoporosis, have largely been based on studies of trabecular bone, the internal network. This is reflected in the degenerated trabecular structure shown in textbook diagrams of osteoporosis. Indeed, the study of trabecular structure, and its control by multiple signaling pathways, has been a useful tool for understanding and treating osteoporosis and other skeletal conditions. Trabecular strength is largely determined by the quantity of trabecular bone present within a given area. The study of trabecular bone's response to potential therapies provides rapid answers because its high surface-to-volume ratio means that small changes in bone formation and resorption can quickly lead to measurable changes in bone volume. The focus on trabecular bone is also historic, since postmenopausal osteoporosis, the most common and most studied form of osteoporosis, was first described in trabecular bone (1). Rodent models, including the ovariectomized rat and mouse, were pivotal for developing current therapies for postmenopausal osteoporosis. Ovariectomized rats were used for the development and testing of bisphosphonates, hormone replacement therapies, and teriparatide in postmenopausal women (although ovariectomy does not mimic the extended period of hormonal variations that occur during the human perimenopause). With the emergence of genetically altered mice, biologics such as denosumab and romosozumab emerged from knockout mouse models showing high trabecular bone mass when RANKL (receptor activator of nuclear factor kappa-B ligand) or sclerostin were removed (2). The first part of this review will discuss the success of mouse models for studying trabecular structure.

A major question remaining is whether mouse models are useful for identifying therapeutic approaches to strengthen the outer envelope of the skeleton, the cortical bone. This must be addressed because the most detrimental osteoporotic fractures occur in weakened cortical bone (eg, at the hip or wrist). These fractures lead to the greatest increases in mortality (3) and morbidity (4) and are the most expensive to treat (5). However, possibly because the field was focused on studying trabecular bone mass, current treatments for osteoporosis, while being very good at preventing vertebral fractures, perform relatively poorly at preventing nonvertebral fractures (6). This is because the strength of vertebrae, which contain a high proportion of trabecular bone, is closely related to trabecular bone mass, while femoral neck and distal radial strength are determined largely by the strength of their dominant component: cortical bone (7).

We will address this question by exploring how the cellular mechanisms and signaling pathways determining bone width, cortical thickness, cortical porosity, corticaosteocyte network integrity, and cortical bone material quality, are conserved between mouse, rat, and human bone, including whether, and to what extent, they are conserved in embryonic and juvenile cortical bone development, degeneration with aging, and their genetic determinants.

## Brief Examples of Rodent Preclinical Models in Development of Current Bone Therapies

Early studies relied on the ovariectomized rat to identify cellular mechanisms by which postmenopausal osteoporosis occurs, and to test therapies now in clinical use. This became well established as a preclinical model of both trabecular and cortical bone loss, including at the femoral neck, in the early 1990s (8-11,). At the time, questions were raised about whether rodent models could be relied on due to their small size, general lack of osteonal bone, short life span, and quadruped nature (12). It became clear, by using sufficiently aged rats and by measuring the correct region (the secondary spongiosa, see “Specialized Terms” box), that ovariectomized rats exhibited bone loss by similar mechanisms to postmenopausal women (12, 13). When used appropriately, the ovariectomized rat has been an excellent predictor of drug effects on bone mass and strength in postmenopausal women (14). Even before postmenopausal osteoporosis was described, in the 1930s, the young intact rat was the model used to first show anabolic (bone-building) properties of parathyroid hormone (PTH) (15), forming the basis of teriparatide therapy (16). Early treatments for osteoporosis, whether antiresorptive bisphosphonates (17-19) or anabolic PTH (20), were proven effective in the ovariectomized rat prior to their clinical use.

Later, genetically modified mice became model systems for discovering novel signaling pathways for osteoporosis treatment, including the osteoprotegerin (OPG)/RANKL system (21). OPG's osteoclast inhibitory action was initially discovered in cell culture studies (22). At the same time, while seeking agents to protect the gut during chemotherapy (21), a second laboratory made the unexpected finding of high bone mass (23) in mice overexpressing the same protein. The absolute requirement of RANKL for osteoclastogenesis, RANK as its receptor, and OPG as its inhibitor were confirmed through later knockout models (24, 25). These mice were pivotal to the development of anti-RANKL therapy (denosumab) (2), now routinely used to treat osteoporosis.

## Mouse Models Recapitulate Human Rare Disease Mutations and Permit Functional Interpretation of Human Genetic Studies

Animal models are used to study the skeletal environment because skeletal development and maintenance are regulated by local and systemic factors and require the interaction between multiple cell types under the influence of mechanical strain and muscular movement. This complexity cannot be modeled in vitro or ex vivo.

Mouse models perform exceptionally well at mimicking human monogenic skeletal disorders. A 2019 review compared 260 genes associated with human skeletal dysplasias, and 96% of the mouse models with the same mutations faithfully reproduced the dysplasia (26). The earliest examples of this were spontaneous mutations, such as the gray-lethal osteopetrotic mouse described in 1935 (27), which led some 60 years later to defining *OSTM1* (Osteoclastogenesis Associated Transmembrane Protein 1) loss-of-function autosomal recessive osteopetrosis (28). Germline and targeted knockouts followed, including the above example of RANKL and RANK deletion which led to osteopetrosis.

High-throughput skeletal phenotyping studies have become more common (29) and are now forming a resource for interrogating genetic variations identified in the clinic, for example, by generating a mouse to reproduce, understand, diagnose, and treat human monogenic conditions. Mouse models for osteogenesis imperfecta are good examples, including the collagen 1 α1 chain G610C mutation (*Col1a1^G610C^*) model, or the less common mutations in interferon induced transmembrane protein 5 (*Ifitm5*), osterix (*Sp7*), and Wingless-related integration site 1 (*Wnt1*) described below. These have been recently reviewed (30). Another example is the use of mouse models to characterize causative genes within candidate loci identified in genome-wide association studies (GWAS) of bone mineral density or fracture incidence (31). Such functional studies often use global knockout or mutant studies (with the same genetic alteration in all cells). Cre-targeting of gene modifications or deletions to specific cell populations or lineages (eg, osteoblast, osteoclast, or osteocyte) is another useful tool; although these do not mimic human conditions, they provide functional information when global deletion leads to early lethality (eg, global parathyroid hormone receptor deletion (32)) or to a confounding systemic defect (eg, global deletion of estrogen receptor leading to high circulating levels of testosterone (33)).

## Key Considerations When Comparing Rodent and Human Bone

### Modeling vs Remodeling During Growth and Aging in Cortical and Trabecular Bone

Bone structure in all vertebrate species with mineralized skeletons is determined by 2 processes: modeling and remodeling. The difference between these processes is the spatial relationship between bone-resorbing osteoclasts and bone-forming osteoblasts. In modeling, osteoblasts and osteoclasts act on different bone surfaces, while in remodeling, the same cells act in sequence on the same bone surface. We describe each in turn.

In modeling, bone formation and resorption are separated by location, and are independent (ie, formation occurs without prior bone resorption, or resorption occurs without subsequent bone formation). Modeling changes skeletal morphology (shape and size). For example, during growth, formation of bone on periosteal (outer) surfaces widens the lengthening bone while bone is concurrently resorbed at a different location (the endocortical surface) to enlarge the marrow cavity. Modeling occurs during the construction and growth of bone, from when skeletogenesis commences in the embryo until longitudinal growth is complete (end of the second decade for human bone) (34). Modeling is also responsible for adaptive changes in bone shape and size in response to increased or reduced mechanical load (35) and with aging (36, 37). Modeling can occur on trabecular surfaces, such as during anabolic PTH action, where some increase in bone mass results from modeling-based bone formation on surfaces without prior resorption (16, 38).

Remodeling is a process of bone replacement by which the skeleton undergoes continuous repair. Remodeling cycles are asynchronous throughout the skeleton at many anatomically distinct sites containing a sequence of cellular players, termed *basic multicellular units* (BMUs) (39, 40). Each BMU follows the same sequence: bone resorption followed by formation. In BMUs, tiny packets of bone are removed by osteoclasts and subsequently replaced at the same location by new collagenous matrix (osteoid) produced by osteoblasts; that matrix is then mineralized to form new bone. Remodeling also contributes to the maturation of trabecular structures from woven to lamellar bone (see below).

**Figure 1.**
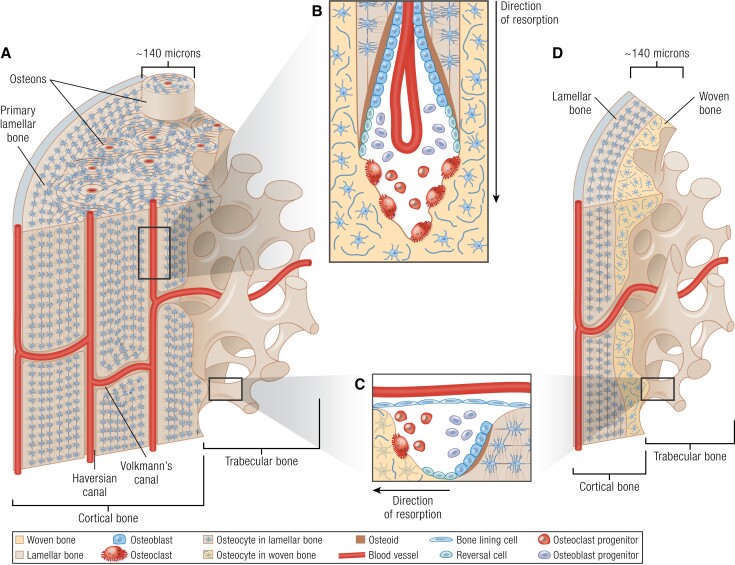
Cortical and trabecular structure (A, D) and remodeling (B, C), in mature osteonal (human, A) and rodent (D) bone. A, In bone of large mammals (including human bone), the cortex contains primary lamellar bone, which consists of circumferential lamellae mostly on the periosteum, but also in some endocortical regions. During early life, this primary lamellar bone is gradually replaced with osteonal (Haversian) bone. Blood vessels oriented perpendicular to the long axis of the cortical bone are termed *Volkmann's canals*. B, During osteonal cortical bone remodeling, cutting cones are formed. Here, the invading blood vessel provides osteoclast progenitor cells, which differentiate into osteoclasts. Osteoclasts initiate a cutting cone by resorbing pre-existing bone, including woven or old, damaged lamellar bone. Osteoblasts follow, filling the excavated canal with new bone (osteoid which becomes mineralized), until the resorbed space is almost filled, leaving a small Haversian canal, and a new osteon. Between the activities of osteoclasts and osteoblasts, reversal cells line the bone surface. C, Surface bone remodeling, including trabecular and endocortical remodeling, which are common to all species, is initiated by release of osteoclast progenitors from blood vessels, which differentiate and resorb pre-existing bone, which may be woven, or lamellar bone. This is replaced with new bone material by osteoblasts; reversal cells cover the bone surface between the activities of osteoclasts and osteoblasts. D, Rodent (mouse and rat) cortical bone, which is thinner than in larger mammals, typically lacks osteons, but like osteonal bone (A), contains lamellar bone closest to the periosteum. In regions closer to the growth plate (top of image), there is a higher proportion of immature woven bone characterized by a less ordered collagen arrangement with a less ordered osteocyte network; this becomes less prominent closer to the diaphysis. Murine cortical bone also contains transcortical, or Volkmann canals, near perpendicular to the longitudinal axis. In murine cortical bone, remodeling occurs on the endocortical and trabecular surfaces, and intracortical remodeling has not been described in physiological conditions. Panel A from LifeART, Lippincott, Williams & Wilkins (2000).

BMUs were first identified based on histology of normal adult human trabecular (39) and human and beagle dog cortical (40) bone, and BMUs are arranged in 2 architectures (Fig. 1). In endosteal bone (including both trabecular and endocortical surfaces), BMUs are located on the marrow-facing bone surface (Fig. 1C) and remodel “pancake-like” packets of bone (41). In osteonal cortical bone (see “Haversian vs Non-Haversian Bone: A Function of Life History”) the BMU comprises cutting cones (Fig. 1B) led by osteoclasts proceeding through bone, digging a microscopic “tunnel”; the osteoclasts are followed by osteoblast precursors which differentiate and refill this tunnel (41). The first BMU microscopic anatomical “products” to appear in cortical bone are termed *primary osteons*; osteons formed by later remodeling cycles are termed *secondary osteons*. This, and nonosteonal cortical bone remodeling, will be discussed in detail below.

For the sequential process of remodeling to occur, osteoclast activity is “coupled” to bone formation by osteoblasts. This means that, in addition to resorbing bone, osteoclasts also produce signals to recruit osteoblast precursors to the resorbed surfaces and to stimulate their differentiation. Such signals have been termed coupling factors, and many have been proposed, acting at different stages of osteoblast differentiation. These proposed factors are reviewed extensively elsewhere (42, 43). When there is an imbalance between resorption and formation, this does not mean the processes are “uncoupled”; the sequence is maintained but the processes are unmatched or out of balance. Regardless of BMU orientation, imbalanced bone remodeling gradually changes bone mass. If bone formation is less than resorption in a majority of BMUs, bone is lost, and if formation exceeds bone resorption, bone mass increases. Systemic biochemical markers of bone formation and resorption do not provide information on whether bone formation or resorption are coupled or balanced at the BMU; these reflect the activities of all bone surfaces in the body, including both remodeling and modeling surfaces.

### A Common Misconception: Isn’t Rodent Bone Continually Growing?

A common criticism against rat and mouse models for bone research is the suggestion that their bones are continually growing. This misconception has arisen because growth plates remain present in rat and mouse bone until at least 12 months of age (44). However, although present, growth plates are largely inactive from 6 months of age onward in both rats and mice (45). This inactivity is seen in histology by the formation of discrete mineralized bony bridges interrupting the cartilaginous growth plate. These expand across the growth plate until it is “sealed” at the metaphysis as a horizontal band of mineralized bone (44). This inactivity is also indicated by growth cessation; in both male and female C57BL/6J mice (the most commonly used inbred strain) femoral length is stable from 6 to 24 months of age (46). This indicates that longitudinal growth has ceased even though the growth plate remains visible at this site. Variation between strains has been reported (reviewed in (47)), and it is likely that anatomical sites would also differ in the timing of when growth ceases. Apart from the work in C57BL/6 femora, very few studies have measured specific skeletal elements with sufficient time points to define when growth has ceased.

The problem of so-called continual growth of rodent models is overstated. In any mouse strain where longitudinal growth continues slowly, regions close to the growth plate would contain new trabecular and cortical bone with a higher proportion of mineralized cartilage and woven bone than mature bone, as outlined below (see “Conserved Development of Trabecular and Cortical Structures During Bone Growth”). Since this region is usually excluded when assessing adult trabecular or cortical bone (see guidelines), there would be no impact of any continuing longitudinal growth on the data obtained.

### Haversian vs Non-Haversian Bone: A Function of Life History

A major structural difference commonly noted between mature rodent and human cortical bone is the lack of osteonal bone in rats and mice. Here we clarify terminology. Remodeling of thick cortical bone, such as human cortical bone, occurs through cutting cones (Fig. 1B), which resorb bone, and are refilled with concentric layers (lamellae) of bone material arranged in cylinders, with a cross-sectional appearance like rings within a tree trunk, around a central vessel (Fig. 1A). The longitudinal pores containing blood vessels are termed Haversian canals. The cylinders of lamellar bone surrounding the Haversian canals are termed osteons.

In evolutionary terms, variation in cortical bone microstructure across different species can be ascribed to variation in mammalian life histories (48). Osteon formation is an ancient ancestral trait; it first emerged in jawed fishes called *Placodermi* about 400 million years ago and has remained through the evolutionary tree in mammals, birds, and some reptiles (49, 50). Both mice and humans therefore have the capacity to remodel their bones, but this capacity is not expressed in the same way across small and larger mammals due to variation in mechanical, physiological, reproductive, and lifespan needs that diversified with evolution. Life history refers to whether an organism evolves “fast-” or “slow-” paced series of biological strategies to optimize energy investment into major lifespan milestones, such as growth, maturation, reproduction, and longevity (51). Slow or fast growth is reflected in mammalian bone vascularization and tissue arrangement and is strongly correlated with body size and longevity (52, 53). Mice, whether wild or laboratory-bred, are smaller and have shorter lifespans than humans, and are thus “fast growers” and their skeletons must form and complete growth quickly. Complex physiological processes seen in larger mammals, such as the formation of osteonal bone, would be costly for small mammals, which is why we see “life history” trade-offs, some of which involve bone metabolic processes (54). As a result, bone tissue in mice and rats is constrained spatially due to their small skeletal size, short life history, and smaller mechanical forces experienced than in larger mammals. This means they have no requirement for the complex remodeling processes developed in larger mammalian skeletons, including humans.

**Figure 2.**
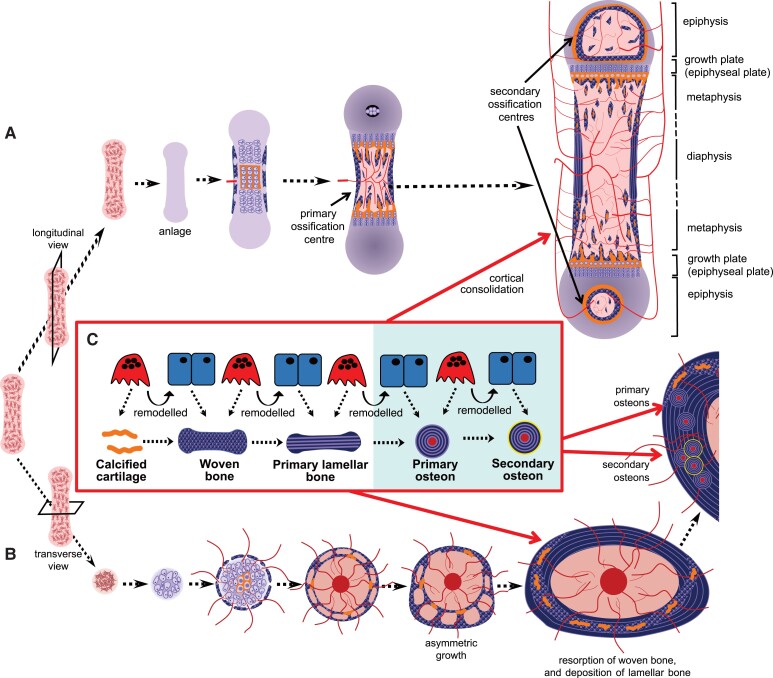
Bone development, viewed as a longitudinal section (A) or a cross-section (B), and the remodeling processes responsible for the transitions in mineralized material (C). During embryogenesis, mesenchymal stem cells condensate and differentiate to form a cartilage model (anlagen) of the bone to be formed. As the bone grows, chondrocytes in the center become hypertrophic and hypoxic, and they release mineral, which accumulates within the cartilage. Blood vessels are drawn to the primary ossification center, bringing osteoclast precursors, which resorb a space into which marrow forms. As the marrow expands, remnants of mineralized cartilage (shown in orange) remain, and woven bone continues to form at the bone collar, forming a porous pre-cortical structure, with infiltrating vasculature. Repeated cycles of remodeling (C) drive cortical bone maturation. Mineralized cartilage is removed by osteoclasts and new woven bone is deposited by osteoblasts. Woven bone is removed by osteoclasts and replaced with primary lamellar bone produced by osteoblasts. In larger species with thicker cortical bone (shaded in blue) further remodeling occurs via cutting cones, resulting in the formation of primary, and then secondary osteons, the latter with cement lines (shown in yellow). A similar process occurs as trabecular bone forms in the primary spongiosa adjacent to the growth plate. A, formation of the primary and secondary ossification centers, and the regions of the immature long bone, including the growth plate (epiphysis), diaphysis and metaphysis. B, the bone grows asymmetrically through the formation of struts and rings of new bone. As the bone continues to grow asymmetrically after birth (modeling), this preliminary bone structure is gradually reshaped; remnants of mineralized cartilage and woven bone are gradually removed and replaced with lamellar bone through remodeling, and cortical drift; some remnants remain into adulthood in the mouse. Panels A and B adapted from (104), with permission.

Haversian systems are not found in human bone at all ages but emerge during bone growth. Mouse, rat, and human bone exist in embryogenesis as a porous woven structure which is initially replaced by more resilient primary lamellar bone (see “Conserved Emergence of Human and Rodent Bone Structure in Embryogenesis”). This primary lamellar bone lacks osteons: it comprises mostly circumferential lamellae (ie, lamellae extending along the bone's circumference, see Fig. 1A). After primary lamellar bone has formed, in larger species, cortical bone undergoes Haversian (cutting cone) remodeling, initially forming primary osteons (Fig. 2C). These are gradually replaced, again by Haversian remodeling, with secondary osteons. Primary and secondary osteons have the same internal structure, but secondary osteons are distinguishable from primary osteons by the presence of cement lines. Haversian remodeling continues until the end of life. This gradual replacement means that the woven bone deposited during human development is rarely seen in the post-cranial bones of human infants past 2 years of age (55). This has been studied in the human humerus, where the first isolated secondary osteons were noted at 2 years of age, and most diaphyseal primary cortical bone had been replaced by secondary osteons after 14 years of age (56). The rate at which secondary osteons appear in the first 2 decades depends on health and lifestyle factors that stimulate remodeling (57). Factors such as poor nutrition can lead to low secondary osteon density, even in older individuals, persisting into the second half of their lifespan (58).

Mature human cortical bone comprises more than osteons and Haversian systems. It also contains primary lamellar bone (lacking Haversian systems) adjacent to the endocortical and periosteal surfaces. One such region in the adult human femur, termed the endosteal lamellar pocket, is likely a “remnant” of diaphyseal cortical drift during ontogenetic modeling (shape changes during growth), and is retained in individuals at least until the fifth decade of life (59). The endosteal lamellar pocket and periosteal lamellar bone lack Haversian systems and osteonal cylinders; they contain only Volkmann's canals, which are semi-perpendicular to the bone's length (Fig. 1A). Through these, the bloodstream is supplied from the periosteal layer to the bone marrow, and egress of marrow-derived hemopoietic cells to the bloodstream also occurs. Haversian systems are most likely required by bones sufficiently large, and with sufficiently thick cortical bone, to require additional vascularization for access of osteoclast precursors to bone distant from the Volkmann's canals. Haversian systems give further mechanical advantage in bones of large mammals, and, perhaps due to increased remodeling, cluster in bone regions experiencing higher strains (60, 61).

The mature cortex of smaller species, such as mouse and rat, contains predominantly circumferential lamellar bone. This exists on the bone's outer and inner cross-sectional thirds, with an (off-center) core of unremodeled endochondral bone and cartilage (see Fig. 1C) (62-64). This has sufficient mechanical competence for a small animal. It somewhat resembles a large osteon and has sometimes been described as a *super osteon* (65). As in human bone, nutrient supply to mature murine and rat cortical bone is provided through a dense vascular system within the bone material (66). What is different between small and large species is the system's architecture: in the mouse, most blood vessels extend through the cortical bone with, generally, a radial cone-like arrangement around the central marrow space, clearly observed in 3 dimensions (3D) by micro- and nano-computed tomography (CT) (67). Perfusion confirmed that these pores contain blood vessels (68). Recent work used the term *transcortical vessels*, which is growing in popularity (69), but since they are perpendicular to the bone length, the pre-existing term *Volkmann's canal* could be more appropriate.

Haversian canals and small osteons have been described in rodent bone. These are typically described in thickened cortical bone regions. Both Haversian canals, oriented parallel to the bone's length (70, 71), and concentric bone layers around those canals, like small osteons (72), have been reported. Their small size is consistent with the concept that osteon size increases in scale with animal size (73). Whether these are large enough to possess radially oriented osteocytes, as seen in larger mammalian osteons, is unknown. However whether radial osteocyte orientation is a defining feature of osteons is questionable since some osteonal bone lacks concentric osteocyte rings: some fish species lack osteocytes yet still exhibit secondary osteons (74). While rodents occasionally have these small osteons, it would be impossible for most adult murine cortical bone (∼140 microns thick (46)) to contain even a single osteon with the diameter observed in adult human bone (∼140 microns in diameter (75)).

In some circumstances, mice also demonstrate intracortical remodeling (bone formation and resorption in sequence within the cortex), although cutting cones have not been observed. In the 1950s, intracortical resorption was induced in lactating rats on a low-calcium diet, and these canals became filled mini-osteons during weaning (76). Intracortical remodeling, marked with calcein labeling, is observed in rats and mice in many conditions. This includes mice with thick cortical bone, such as the C3H strain (77, 78), sclerostin deletion (79), aging (80-82), ovariectomy (83), induced renal disease (84-86), and the range of genetic modifications discussed below and outlined in Table 1. This means that rodents have potential for intracortical remodeling, even though, in the most common strains with very thin cortical bone, this potential is only reached when there is direct experimental stimulation.

**Table 1.** Mouse models with high cortical porosity and, where appropriate, human equivalents

Experimental model	Age and sex of mice reported	Bone resorption	Bone formation	Vascularity	Woven bone	Cortical thickness	RANKL/OPG	PTH	Human equivalent
**Genetic strain**									
C3H strain compared to C57BL/6 (78)	26-week-old; female	↑	↑BFR	—	↑	↑	↔	↑	
**Aging**									
Aging, C57BL/6 (80)	18 and 26-month-old; female	↑	↔	—	—	↓	↑RANKL ↓OPG	—	✓(87, 88)
Aging, C57BL/6 (81)	1 to 26 month-old; male	—	↓	—	—	↓	—	—	
Aging, C57BL/6 (82)	5- and 22-month-old; female	↔	—	—	—	↓	—	—	
**Ovariectomy**									
Ovariectomy, C3H (83)	32-week-old female	↑	↓	—	—	—	—	—	
**Gorham-Stout disease**									
Postnatal overexpression of VEGF-C in osteoblast lineage for 35 days (89).	35-day-old; sex not reported	↑	—	↑ lymphatics	—	↓	—	—	✓ (90)
**Chronic kidney disease models**									
0.2% dietary adenine for 10 weeks (84).	25-week-old; male and female	↑	↑BFR	—	—	↓	—	↑	✓ (91)
Partial renal ablation with high dietary phosphate for 12 weeks (85).	30-week-old; female	—	—	—	—	↓	—	↑	
0.2% dietary adenine for 6 weeks (86).	78-week-old; male	—	—	—	—	↓	—	↑	
**Modified Wnt signaling**									
Global WNT16 knockout (92).	5 and 11-week-old; female	↑	—	—	—	↓	↓OPG	—	
WNT16 knockout in osteoblasts and osteocytes (92).	5-week-old; female	—	—	—	—	↓	↓OPG	—	
Sclerostin knockout mice (79).	10-16-week-old male, 12-week-old female	—	↑ BFR	—	—	↑	—	—	
**Cathepsin K activity modulation**									
Global Cathepsin K knockout (93).	10-week-old; male and female	↓	↔ BFR endocortical	—	↑	↑	—	—	
Cathepsin K overexpression in osteoclasts (94, 95).	1 and 3-month-old; female	↑ Cathepsin K & TRAP	↔ BFR	—	—	↑	—	—	
**VEGF modulation**									
VEGF overexpression for 2 weeks (96).	3-4-month-old; male and female	↑	↑only woven bone	↑	↑	—	↑ RANKL	—	
**Glucocorticoid administration**									
Slow-release glucocorticoids for 28 days (97).	8-month-old; male	↑	—	—	—	↓	↓OPG	—	
**RANKL/OPG manipulation**									
Twice-daily RANKL injections for 10 days (98)	11-week-old; female	↑	↑Periosteal BFR	—	—	↓	↑RANKL	—	
RANKL overexpression (99)	3 and 8-month-old; male and female	↑	—	—	↑	↓	↑RANKL	—	
Global OPG deletion (25)	1-week and 2-month-old; female	↑	↑	↑	↑	—	↓OPG	—	
GATA-1 knockdown in OPG-deficiency (100).	5-month-old; female	↑	—	—	↑	↑ cross-sectional area	↓OPG	—	
**Elevated local STAT3 signaling**									
SOCS3 ablation in osteocytes (101-103).	12 and 16-week-old; male and female	↑	↑	↑	↑	↔	↑RANKL(142)	—	
SOCS3 ablation in osteocytes with IL-6 knockout (102).	12-week-old; male and female	—	—	—	↑	—	—	—	
SOCS3 ablation in osteocytes with global G-CSFR deletion (104).	6, 12 and 26-week-old; male and female	↑	↑	↑	↑	↑	↑RANKL	—	
SOCS3 and leptin receptor ablation in osteocytes (105).	6 and 12-week-old; male and female	↔	↔	—	—	↔	—.	—	
**PTH administration or receptor activation**									
Constitutively active PTH1R in osteoblast lineage (106)	6-month-old; male	↑	↔	↑	—	↑	—	↑PTH1R	✓ (107)
2 weeks continuous PTH infusion (108).	10-week-old; male and female	↑	↑	—	—	—	↑RANKL	↑	
Intermittent PTH injections in sclerostin-null mice (79)	10-16-week-old male, 12-week-old female	—	↑	—	—	—	—	↑	
Knockout of PTH and PTHrP (109).	Newborn; sex not reported	↓	↓	↓	—	↑	—	↓	
Knockout of PTHrP (109).	Newborn; sex not reported	↓	↑	↑	—	↑	—	↑	
**Diabetic models**									
Streptozotocin-induced hyperglycemia (110).	10-11-week-old; male	—	↔	—	—	↑	—	↑	✓ (111)
10 weeks high fat diet induced type 2 diabetes mellitus (112).	16-week-old; sex not reported	—	↑	—	—	↔	—	—	
Streptozotocin-induced diabetes with β-catenin activation in osteoblasts (113).	14-week-old; male	↑	↓	—	—	↓	↑RANKL	—	
**Other models**									
Bone sialoprotein (BSP) knockout (114)	2-month-old; male and female	—	—	—	—	↓	↑RANKL	——	
Double knockout of BSP and osteopontin (114)	2-month-old; male and female	—	↔	↔	—	↔	↑RANKL	—	
Fatigue loading for 15 days (115)	3-month-old; male	—	—	—	↑	↑	—	—	
Compound hemizygous mice for *Gja1* (Connexin 43) and *Runx2* (116, 117).	8-week-old; male	↑	—	—	—	↔	↑RANKL to OPG	—	
Plastin-3 global knockout mice (118).	6, 12 and 24-week-old; male	↑	↔ (BFR)	—	—	↓	—	—	
Midkine-deficient mice (119, 120).	18-month-old; female	↑	—	—	—	↔	↓RANKL, ↔OPG	—	
Colony-stimulating factor-1 (CSF-1) overexpressed in osteoblasts (121).	14-week-old; male	↑	↑	—	↑	↑	—	—	
Bone morphogenetic protein 1 receptor knockout in osteoblasts (122)	9-week-old; male	↔	↔	—	—	↔	↔	—	
PPARγ deletion from early osteoblast progenitors (123)	22-month-old; female	↔	↔ (BFR)	—	—	↔	—	—	
Mice with osteocyte-specific ablation (124)	10-week-old; male	↑	↔ (BFR)	—	—	—	↑RANKL	—	

Shown are examples of mice with high cortical porosity, grouped thematically, with details about age and sex of mice, and other changes described in the cortical bone, in circulating parathyroid hormone (PTH) or PTH/PTHrP receptor expression (PTH1R). See text for further details.

Legend: ↔ No change, — Not reported.

Abbreviations: BFR, bone formation rate; OPG, osteoprotegerin; RANKL, receptor activator of nuclear factor kappa-B ligand; SOCS3, suppressor of cytokine signaling 3.

## Conserved Emergence of Human and Rodent Bone Structure in Embryogenesis

We now turn to the extent to which bone development is conserved between species during embryogenesis, prior to osteon formation. The 2 processes that form the preliminary structures of cortical bone—intramembranous ossification and endochondral ossification—are highly conserved between species.

In all mammalian species, cortical structures in the flat bones, such as the skull, mandible, maxilla, and clavicles form by intramembranous ossification. In this process, stromal progenitors accumulate and differentiate directly into osteoblasts, then deposit collagen I–containing osteoid, which is mineralized to form bone. Flat bones grow by osteoblast differentiation at the periphery (periosteal surfaces and calvarial sutures). The processes controlling flat bone growth and calvarial suture closure are highly conserved between rodents and human primates, indicated by excellent concordance of murine skull phenotypes with human cranial dysplasias (26). Since these structures do not determine fracture susceptibility, their differences between mouse and human will not be discussed here.

Most bones form largely by endochondral ossification. This is illustrated from 2 perspectives in Fig. 2, with panel B highlighting the cross-sectional appearance at the diaphysis. Like intramembranous ossification, endochondral ossification is also initiated by stromal cell condensation, but an intermediate cartilaginous (chondrocyte) template is formed before bone formation commences. This cartilage template enlarges through chondrocyte proliferation and cartilage matrix production, which gradually transforms into a larger mineralized bone by 2 processes: bone collar formation and ossification center formation. Ossification centers form when chondrocytes become hypoxic, undergo hypertrophy (enlarge), and mineral accumulates in the surrounding cartilage matrix. This mineralized cartilage is invaded by blood vessels, which bring osteoclast and osteoblast precursors (125). Subsequent osteoclast formation leads to mineralized cartilage resorption, making space for blood vessels and expanding the marrow cavity; the first region of vascular invasion and marrow formation within each mineralized template is the *primary ossification center*. Differentiated osteoblasts form osteoid on the remnant cartilage templates, which mineralizes to form bone.

In embryonic murine (126) and human (127) long bones, the nascent diaphyseal and metaphyseal cortical structures are highly porous, vascularized, and barely distinguishable from trabecular bone, other than by their location at the bone's periphery. During these early developmental stages, collagen is deposited rapidly with an irregular orientation and is called *woven bone*. This porous precursor to the diaphyseal cortex, termed the *bone collar* or *ring of Lacroix*, is formed by osteoblasts with vascular invasion of the perichondrium, the cellular condensation surrounding the cartilage template (128). Cortical and trabecular structures begin to emerge during this earliest stage of bone development. The diaphyseal cortex widens through periosteal bone modeling, and both human (127) and murine (126) cortices display alternating struts and rings of woven bone, interspersed with blood vessels and marrow. After birth, the highly porous woven bone precursor to cortical bone is remodeled to form a dense, more mechanically competent, layered structure, which lacks the larger marrow-containing spaces observed in neonates but retains an infiltrating vascular structure in human (127, 129) and mouse (62, 130). This high vascular porosity persists in the human femoral cortex during the first 2 decades of life, reaching a minimum when peak bone mass is achieved (from age 20-30 years) (131).

**Figure 3.**
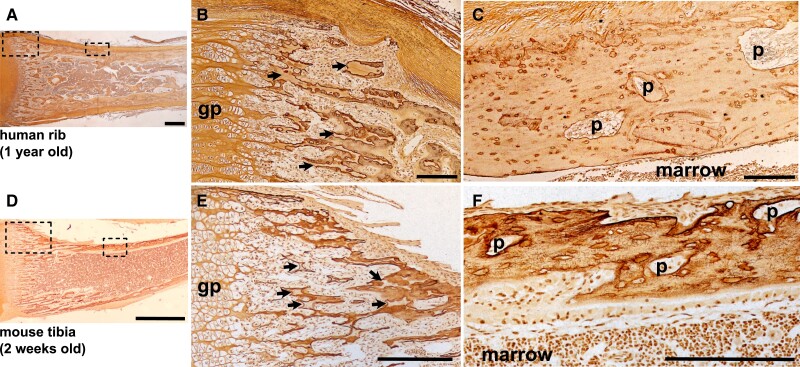
Comparative histology of metaphyseal consolidation in human rib (A-C, 1 year old male) and murine C57BL/6 tibia (D-F, 2 week old male) stained by Ploton silver. Low power images (A, D) show a morphological overview of the metaphyseal region (scale bar = 1 mm); dashed boxes show regions highlighted in panels B, C, E, F. Panels B (human) and E (mouse) show the peripheral metaphysis immediately adjacent to the hypertrophic zone of the growth plate (gp) showing extensive unremodeled cartilage remnants (black arrows) in the newly deposited trabecular and cortical bone of both species. Panels C (human) and F (mouse), show more mature cortical bone, lacking lamellar osteocyte organization, and containing cortical pores (p) in both species; scale bar = 200 micron.

The process of cortical development in the metaphysis differs from the diaphyseal bone collar. As the cartilaginous growth plates move apart from each other during longitudinal growth driven by hypertrophic enlargement of chondrocytes, remnants of mineralized cartilage are used as templates on which both cortical and trabecular bone form in the metaphysis (132). As the bones grow, through cycles of bone remodeling, the mineralized cartilage is gradually replaced with woven bone, which is then replaced by lamellar bone. In the embryo, particularly the murine embryo, the small amount of trabecular bone present in the primary spongiosa is largely mineralized cartilage and woven bone. In both the cortex and the trabecular bone, remnant mineralized cartilage and woven bone remain until remodeled into a more mature structure in both human and mouse (Fig. 3) (62, 133).

In smaller bones lacking growth plates, such as the tarsals and metatarsals, the process of cortical bone development still follows the endochondral ossification program, and is similar to cortical development in the epiphyses, since both lack the longitudinal growth and columnar organization of the growth plates. This process too, is conserved between mouse and human (134).

## Conserved Development of Trabecular and Cortical Structures During Bone Growth

### Development of Trabecular Structures in Human and Murine Bone

Trabecular bone arises from the growth plate, and in primary and secondary ossification centers, through targeted remodeling. The first remodeling sequence is when mineralized hypertrophic cartilage is resorbed by osteoclasts, provided by the vasculature, followed by osteoblasts acting on the resorbed surface to deposit woven bone. Next, the woven bone is resorbed and replaced with lamellar bone in a second cycle of remodeling (135). This is remodeling because it is BMU-based resorption followed by bone formation on the same bone surface. Although it is the same sequence of events as adult trabecular remodeling, it differs in 2 respects: (i) it replaces one type of substance with another; and (ii) the processes have a specific anatomical location relative to the growth plate. Since it occurs in defined anatomical regions, it is likely subject to different control mechanisms than remodeling in the adult trabecular network.

This region-specific control of osteoclast and osteoblast activities is extremely challenging to study in humans, given the lack of specimen availability. However, as a comparative example, in patients with Stüwe-Wiedemann syndrome, caused by loss-of-function mutations in the receptor for leukemia inhibitory factor (LIF) (136), growth plate defects have been observed. These are very closely mimicked in mice lacking the ligand (137), where the increased resorption was specific to the primary spongiosa, due to a region-specific increase in vascularization driven by elevated expression of vascular endothelial growth factor (VEGF) at the growth plate (137).

This targeted remodeling in the primary spongiosa must be kept in mind when studying rodent bone. Unlike human biopsies, studies of rodent bone usually use the entire tibia or femur, and the growth plate and primary spongiosa are present in the histological section. Since murine tibiae and femora have so little trabecular bone, many published studies measure histomorphometry at the primary spongiosa because this provides plentiful bone surface. However, this region reflects the site-specific remodeling that generates trabeculae, and differs from adult bone remodeling. This is illustrated in LIF-deficient mice (137), and in mice with collagen mutations resistant to collagenases (138) or aggrecanases (139) or with reduced ADAMTS4 (a disintegrin and metalloproteinase with thrombospondin motif 4) production due to EphrinB2 deficiency (140). All these defects led to transient osteopetrosis in the first few days after birth due to defective targeted remodeling of growth plate cartilage, but normal resorption on non-targeted remodeling bone surfaces in the secondary spongiosa of older mice (141).

Extrapolating data from the primary to the secondary spongiosa was identified as a problem in early studies of the ovariectomized rat (10, 11). The guidelines developed then also apply to genetically modified mice: if studying trabecular bone remodeling, measurements must be made in the secondary spongiosa, in regions where lamellar bone is remodeled, after longitudinal growth has slowed. If pharmacological treatments are being tested, the region must be sufficiently far from the growth plate to have already contained secondary spongiosa when treatment commenced. This is necessary to avoid confusing a growth-related or region-specific effect with an effect on adult bone remodeling. This is outlined further in the guidelines below.

### Development of Cortical Bone at the Metaphysis Requires Sufficient Chondrocyte Maturation, Vascularization, and Bone Resorption

After the cortical structure is initially established at the diaphysis, cortical bone continues to form at the metaphyses during longitudinal growth through cortical consolidation: trabeculae at the periphery, which emerge from the growth plate, coalesce into a thickened cortical structure (142). As at the diaphysis, the bone is transformed from a porous, trabecular structure to a compressed cortical structure containing whorls of compressed trabecular bone, and then through remodeling, this structure is gradually converted into lamellar bone. We use the term *porosity* or *cortical porosity* here to refer to microscopic pores, usually containing blood vessels and sometimes marrow. There are other porosities in the cortex, including *lacuno-canalicular porosity* which describes smaller pores occupied by osteocytes and their dendritic processes and a subcanalicular *nano-porosity* network, very recently been described in human bone specimens (143) but not yet studied in mice.

The emergence of the cortex from the growth plate is morphologically similar in human and murine bone (101, 144). Histologically, close to the active growth plate, both exhibit sparse, disorganized cortex containing seemingly randomly oriented, poorly connected osteocytes, a high proportion of cartilage, many intracortical pores and cement lines, and extensive periosteal resorption (Fig. 3). Reflecting this process of maturation, both human and murine bone exhibit a gradient of mineral density along the metaphysis, with a gradual reduction in cortical porosity and increase in matrix mineralization with increasing distance from the growth plate (101, 145).

When endochondral ossification is delayed, so too is cortical development. For example, when parathyroid hormone related protein (PTHrP), a cytokine required for endochondral ossification, was deleted in a mouse model, the bones were shorter than normal. In addition, the cortical bone was immature and more porous than in the wild type (109), reflecting delayed bone consolidation within the developing embryonic cortex due to delayed primary ossification center formation.

The processes shaping the cortical and trabecular compartments depend on appropriate resorption and formation at specified locations. *Osteopetrosis*, an umbrella term for the high bone mass conditions caused by low bone resorption, illustrates the importance of osteoclasts in this process. In osteopetrosis, bone resorption is impaired, either due to deficient osteoclast formation or deficient osteoclast function. The high bone mass of osteopetrosis emerges during embryonic bone development, preceding formation of the trabecular and cortical compartments. Calcified cartilage resorption is impaired, and ossification center and subsequent marrow space formation is delayed. In human osteopetroses, the marrow space retains thin, unremodeled trabecular structures with abundant woven bone, but little lamellar bone, and unresorbed calcified cartilage remains within the trabecular bone even distant from the growth plate (146, 147). These features are also observed in murine osteopetroses (24, 148, 149). Since the entire femur and tibia can be studied in mice, cartilage remnants within trabecular bone are a useful and quantifiable marker of impaired growth plate resorption, even in mild forms of osteopetrosis (150, 151).

Individuals with osteopetrosis also exhibit delayed cortical bone development, with persistence of woven bone and cartilage remnants, and few Haversian systems (146). Few images have been published, due to the difficulty of obtaining cortical bone samples. In humans, biopsies are rarely available, and even examination by high-resolution peripheral quantitative CT (HR-pQCT) is challenging to interpret. A recent study reported thick cortical bone in a single patient, but the cortex was indistinguishable from the dense, non-remodeled trabecular structure (152). A delay in cortical bone development is readily observed in mouse models, where the whole bone can be examined histologically and compared to matched controls. For example, in the c-*src* null mouse, which lacks osteoclasts, the cortex is thin and remains highly porous, even in the diaphysis, indicating a lack of cortical consolidation (148). Even in mild resorption defects, (eg, the Cathepsin K null mouse), high cortical porosity is observed in early adulthood (153). Initially this seems counterintuitive: why does osteoclast inhibition cause increased porosity when osteoblasts would be responsible for filling the porous cortical structure? It suggests cortical consolidation is driven by remodeling: the osteoblasts responsible for closing cortical pores must depend on prior resorption by osteoclasts. This concept is supported by delayed bone collar formation and delayed cortical consolidation in mice lacking VEGF isoforms, with reduced osteoclast invasion due to defective vascular formation (154). In contrast to the intracortical environment, *src* null mice exhibit normal periosteal diameter (148) confirming that bone width during embryogenesis is determined by modeling and is independent of osteoclast function.

In addition to their high trabecular bone mass, osteopetrotic mice and humans also exhibit an Erlenmeyer flask morphology (sometimes termed Erlenmeyer flask deformity). This is readily observed by x-ray, and characterized by a wider than normal metaphysis which lacks a concave connection to the diaphysis; it is usually accompanied by cortical thinning (155-157). The lack of a concave shape in osteopetrosis shows the importance of periosteal osteoclast-mediated bone resorption in both human and murine bone. Mouse models lacking osteoclasts, like the RANKL or M-CSF (macrophage colony stimulating factor) knockouts, exhibit Erlenmeyer flask morphology on x-ray (24, 149). So too, do mouse models generated to mimic known human osteopetrotic mutations. One example is the human osteopetrosis caused by SNX10 (sorting nexin 10) deficiency, where osteoclasts are nonresorbing (158). Introducing the mutation into mice also resulted in osteopetrosis with a nonconcave metaphysis, revealing a new mechanism controlling osteoclast activity (157). Erlenmeyer flask deformity is not limited to osteopetrosis in human syndromes (156), and this morphology can be observed, with varying ranges of severity, in knockout and mutant mouse models (137, 159-162). These have the potential to reveal new insights about genes responsible for metaphyseal development, but they are rarely reported as there is no standard way of quantifying this phenotype in human or mouse. We recommend reporting metaphyseal bone widths in both anteroposterior and mediolateral directions at defined points from the growth plate to standardize this and lead to new discoveries.

### Closure of Cortical Pores at the Metaphysis Requires Sufficiently Low Bone Resorption

A second situation in which the distinction between cortical and trabecular bone is disrupted is when levels of bone resorption remain high, and the porous cortex does not consolidate. This has been described in a range of mouse models (Table 1) but not in human bone. This may be because the latter would require histological examination of the metaphysis in adolescent individuals with rare genetic conditions, where diagnosis is usually limited to x-ray analysis.

One example of this in mice is when RANKL was overexpressed in bone cells normally expressing this cytokine, including osteoblasts, osteocytes, and hypertrophic chondrocytes (99). In this study, cortical porosity was elevated, and there was a greater proportion of woven bone within the cortex. In the context of elevated bone resorption, the presence of woven bone suggests an inappropriately high level of bone remodeling, with rapid bone deposition, akin to fracture healing. This indicates that a sufficiently low level of resorption is required for cortical consolidation to occur. Two studies of genetic OPG deficiency in mice throughout life also observed high cortical porosity with increased osteoclast number and activity, as well as a high proportion of woven bone (25, 100). This is also illustrated in human conditions with elevated RANKL activity, which show extensive osteolysis and the aberrant presence of woven bone (163). In this way, the replacement of woven bone with lamellar bone during cortical consolidation, in both the mouse and the human, requires a sufficiently low level of osteoclast activity.

Osteoclast progenitors are supplied to the cortex through the intracortical vascular network, which forms as the new cortex is developing during embryogenesis. This has been illustrated by studies of VEGF, a pro-angiogenic factor. VEGF overexpression in osteochondroprogenitor cells delayed cortical development during embryogenesis. There were more blood vessels within the porous cortex due to activated VEGF receptor-2 signaling in both osteoblast lineage and endothelial cells (96). These vascular pores were lined by osteoclasts, suggesting the high level of angiogenesis in the porous early-stage cortex enabled elevated migration of osteoclast precursors to the intracortical bone remodeling surfaces (164).

Even after the cortex has reached its adult shape, the consolidation of cortical material continues, with compressed trabecular bone and woven bone being replaced, by bone remodeling, with lamellar bone (Fig. 2). This process is similar to the development of trabecular structure: cycles of remodeling replace the cartilage remnants with woven bone, and then lamellar structures (165). In murine and human cortex, this includes cortical drift (see “Cortical Drift Shapes Rodent and Human Cortex in Response to Mechanical Loads”) (Fig. 2) (101, 142).

If levels of bone resorption are too high after the initial stages of bone development, excessive intracortical remodeling still delays its consolidation, due to continuing formation of woven bone. This was demonstrated in mice with increased STAT3 (signal transducer and activator of transcription 3) signaling in osteoblasts and osteocytes, generated by deleting the STAT3 inhibitor SOCS3 (suppressor of cytokine signaling 3) in *Dmp1Cre-*expressing cells (mature osteoblasts and osteocytes) (101, 102). In these mice, a high level of intracortical resorption continued into adulthood, thereby suppressing cortical consolidation; the mice retained a porous and highly woven whorl-like structure of compressed trabecular bone until early adulthood (101). Even when these mice were aged until the cortical structure was fully established, the mice with delayed corticalization had weaker bones (101, 102). This suggests that delayed cortical consolidation may reduce strength in the mature skeleton. Indeed, in human puberty, cortical consolidation is impaired during periods of very rapid longitudinal growth (166). Studies of mother/daughter pairs indicate that this may contribute to fracture risk later in life (167).

The phenotype of this SOCS3-deficient mouse, and a related mouse where deletion was targeted to chondrocytes and osteoblasts (168), indicated that STAT3 signaling in bone must be suppressed for cortical bone to transition from compressed woven bone to lamellar bone. These SOCS3-deficient mice also provided evidence of intracortical remodeling in adult mice, albeit nonosteonal. When granulocyte colony-stimulating factor receptor (G-CSFR) was genetically ablated in the SOCS3-deficient mice, the intracortical remodeling was exaggerated even further, with consolidated cortical bone still lacking in 6-month-old mice (104). Further analysis revealed an inverse relationship between osteocytic STAT3 phosphorylation and cortical consolidation: higher levels of STAT3 phosphorylation in osteocytes were associated with greater levels of RANKL, higher levels of osteoclast formation, and a greater delayed in cortical consolidation; this explained why the female mice of this strain had a more severe phenotype (104, 105). In addition to having higher levels of bone resorption in the cortex, the mouse models with highest levels of STAT3 phosphorylation also had greater intracortical vascularization and greater levels of VEGFA expression within the cortex (104).

These delayed cortical consolidation models indicate that murine bone, like human bone, undergoes intracortical remodeling during its development, since intracortical pores contained both osteoclasts and osteoblasts within a woven structure (101). These osteoclasts likely emerge from the transcortical channels/Volkmann canals and are under the influence of interleukin (IL)-6 family cytokines, a group of cytokines known to stimulate STAT3 and both osteoblast and osteoclast formation (reviewed in (169)).

Since clinical imaging methods lack the resolution to describe developmental defects in cortical consolidation in human bone, mouse models are needed to study the contributors to this process. It is not relevant that murine intracortical remodeling during consolidation is nonosteonal since these processes occur prior to the emergence of primary and secondary osteons and are therefore also nonosteonal in human bone. The transition from woven bone to whorls of compressed trabecular bone is conserved between species, and the signals controlling this can be studied in the mouse.

### Cortical Drift Shapes Rodent and Human Cortex in Response to Mechanical Loads

Bone growth is asymmetrical in both longitudinal and transverse directions. Cortical drift is the process by which bones change their shape along the transverse axis during growth. This occurs in both human and murine bone as an adaptation to the mechanical loads and muscle forces associated with movement. This is most striking in the tibia, where bone formation on the medial periosteal and lateral endocortical surfaces changes symmetrical shapes during early embryonic development to (for example) the curved tibia seen in adulthood. It is also apparent in the femur, which becomes more elliptical during early postnatal life in human (170) and rodent (126).

The nature of this drift can be readily appreciated in thick transverse sections of both human and murine bone (59, 62, 63). Both exhibit a core of immature bone, including highly mineralized remnant calcified cartilage, within the most stable and oldest region of lamellar bone on the anterior and medial sides. It is also clearly indicated by the presence of primary lamellar bone on periosteal and endocortical surfaces in transverse sections in human bone (170). In experimental models, the drift can be observed through repeated administration of fluorochrome labels, where deposition on opposing cortical surfaces can be readily appreciated (171). In rat and mouse models, this pattern of growth and the region of measurement must be considered, and if required, controlled for when carrying out histomorphometry of either endocortical or periosteal surfaces.

### Defects in Cortical Thickness and Bone Width Conserved Between Mouse and Human

We have recently reviewed murine models with abnormal bone width and cortical thickness, many of them with related human conditions (172). Here, we will give one key example, which has been extensively studied through multiple approaches, and direct the reader to our earlier review for further examples.

Secreted frizzled related protein 4 (sFRP4) was recently discovered as a Wnt signaling inhibitor determining normal cortical thickness. Pyles disease, a metaphyseal dysplasia, characterized by widened metaphyses, Erlenmeyer flask morphology and thin cortical bone is associated with loss-of-function mutations in the human *SFRP4* gene (161). High trabecular bone mass, widened metaphyses and a wider, more porous femoral neck were also described in *Sfrp4* null mice (160-162), confirming sFRP4's functional importance in bone metabolism. The mechanisms by which these bone defects arose were difficult to understand from clinical investigations since the patients exhibited normal serum markers of bone resorption (161). However, in the mouse model, a region-specific increase in osteoclast formation on the endocortical surface was measured and found responsible for the reduced cortical thickness (161). Again, as in the patients, this was not reflected in serum markers of bone resorption, which were normal. The cause of cortical thinning could not be discovered by studying the human pathology but was readily identified in the mouse model.

This provides an example of how mice, despite lacking osteonal bone, recapitulated a human condition with thin cortical bone, and helped understand the human pathology. Mouse models of human monogenic conditions with thick cortical bone also demonstrate thick cortical bone (eg, LRP5 high bone mass mutation (173), Van Buchem disease, or sclerosteosis (174)). Other examples are included in our earlier review (172) and show that cellular activities on the periosteal and endocortical surfaces determine cortical bone thickness and bone width, are conserved between species, and do not depend on the existence of osteonal remodeling or anatomy.

### Collagen Deposition and Mineralization Control the Osteocyte Network in Trabecular and Cortical Bone Development, Osteogenesis Imperfecta, and Rickets

During bone formation, osteoblasts are incorporated into the newly deposited bone matrix and differentiate into osteocytes (175) which form an extensive interconnected cellular network through the bone structure (176). The nature of this network and the shape of the lacunae in which the cell bodies reside depends on the type of bone deposited. In woven bone, osteocytes have larger and more rounded cell bodies, with shorter dendrite-like processes, and lack any clear pattern of orientation (recently reviewed in (177)). In contrast, in lamellar bone, where collagen is deposited in layers, osteocyte cell bodies are ellipsoid, flattened along the lamellae, and their dendrite-like processes preferentially extend through the lamellae, being mostly oriented perpendicular to the surface on which bone was deposited (178, 179).

Osteocyte network structure differs with bone tissue age. During skeletal growth, this is driven by the replacement of woven bone with lamellar bone by targeted remodeling. It can be readily observed in murine cortical bone from mice of different ages, where the young femoral diaphysis (eg, 3 days to 8 weeks) contains a high proportion of woven bone (165). This is gradually replaced by lamellar bone, and older cortex (from 12 weeks of age) exhibits lamellar bone, with strongly oriented osteocyte lacunae and canaliculi (165).

This difference in osteocyte network architecture can also be observed along the length of individual bones in both human and murine histology. The initial woven bone structure in the trabecular primary spongiosa is replaced with newly deposited lamellar bone (Fig. 3). In human and murine cortex, woven bone is also gradually replaced with lamellar structures (101, 142). In both instances, cell body orientation, canalicular, and dendritic structures are determined largely by whether the collagen substrate into which the osteoblasts are embedded is woven or lamellar (180).

The osteocyte network has been more commonly investigated in murine bone than in human, due to the ease of obtaining specimens (Table 2), particularly in rare genetic conditions. The network structure is assessed by multiple methods—most, like the Ploton silver stain (198), Bodian stain (165), and Rhodamine G infiltration of bone samples (199), visualize the canalicular network, rather than the dendrite-like processes of the cells themselves. In contrast, studies using intracellular stains for actin (eg, phalloidin) (200) or genetically introduced green fluorescent protein (GFP) tags (201) visualize the cells themselves. The 2 types of methods should not be directly compared, because canals do not always reflect the extent of dendritic processes which are motile and extend and retract along the canaliculi (202).

**Table 2.** Mouse models with osteocyte network defects, grouped by the nature of the defect

Genetic disruption/treatment/process	Age and sex of mice	Cell bodies or lacunar changes	Canalicular changes	Dendritic changes	Collagen defect	Mineralization defect	Cortical phenotype	Other changes
**Aging**								
Aging (181)	36-month-old; male	↓ osteocyte density	↑ tortuosity	↓ number of processes				
Aging (182)	3-, 6-, 12-, and 24-month-old mice; male	↓ osteocyte density		↓ total process length / cell				
Aging (82)	5-, 12-, 18-, and 22-month-old; male and female	↓ cell volume and density; “islands” of disconnected osteocytes	↓ canaliculi per lacuna; ↑ gaps in network	↓ connectivity, ↓ processes		regional variation in mineral density	↑ cortical porosity, ↓↓ cortical thickness	↑ empty lacunae
**Changed canalicular network**								
*Ifitm5* gene mutation (71)	4- and 8-week-old; female	↑ cell density	↓ canaliculi			↑mineralization		
Global dentin matrix protein 1 (DMP1) deletion (183, 184).	2- to 5-month-old mice; sex not reported	↑ lacunar size, random orientation	↓ canaliculi	↓ number of dendrites	unmineralized collagen fibrils	diffuse, patchy mineralization (184); ↑ osteoid		↔apoptosis
Global and osteocyte-targeted *Plex* knockout (185).	7-week-old; male and female	↑ lacunar size, random orientation				↓mineralization		lacuno-canalicular wall buckled and enlarged
Deletion of Yes-associated protein (YAP) and transcriptional co-activator with PDZ-motif (TAZ) in osteoblasts and osteocytes (186).	4 and 12-week-old; male and female		↓canalicular density and process length	↓branch length, number of branches and number of junctions per cell	↓collagen content and organization		↓cortical thickness,	↓metalloproteinase and cathepsin K levels in bone
Transforming growth factor beta receptor I (TβRI) inhibition (187)	11-week-old; male		↓canalicular length					↓metalloproteinase (*Mmp2*, *13*, *14*), cathepsin K and tartrate resistant acid phosphatase
Ablation of TGFβ type II receptor (TβRII) in osteocytes (181, 187)	2-month-old; male	↔ osteocyte density, ↔ lacunar volume, shape, orientation	↓canalicular length and density, ↑ canalicular tortuosity	↓number of dendrites		↓mineralization	↔ cortical thickness	↓ metalloproteinase (*Mmp2*, *13*, *14*), cathepsin K and tartrate resistant acid phosphatase
Global matrix metalloproteinase 2 (MMP2) deletion (188)	3-, 11-, and 55-week-old; sex not reported		↓ connections between adjacent lacunae, ↓ processes			↓ mineralization ↔ endosteal and periosteal mineral apposition rate		↑ ratio of empty lacunae in calvariae only and not in long bones
Global membrane type-1 matrix metalloproteinase (MT1-MMP) deletion (189)	20-day to 70-day-old; sex not reported	↔ osteocyte viability, density, and number of osteocytes	↓ number of processes, process length		↓ collagen fibrils degradation			↔ apoptosis
**Osteocyte maturation and survival**								
Inactivation of von Hippel-Lindau gene (*Vhl*) in mature osteoblasts and osteocytes (190).	6-week and 8-month-old mice	↓ diameter and volume; cell bodies thinner and longer	↓ canalicular number and density; random arrangement		↓ collagen organization			↑ empty lacunae, cytoplasmic shrinkage, apoptosis
*Col1a1^r/r^* mice with collagenase-resistant type I collagen (191).	3-week-old; sex not reported		↓ connections between adjacent lacunae; ↓processes		↓ cleavage of collagen α1/α2 chains			↑ empty lacunae (191)
Homozygous *Ankrd11* deletion in neural crest cells (192).	neonates; sex not reported	↑ osteocytes; plump cells, irregular distribution			↑ collagen fibril cross-linking			
Global macrophage colony-stimulating factor (CSF-1) knockout (149).	3-week-old; sex not reported	Irregular shape, random distribution		poorly defined dendrites	↓ collagen fibril length and number; woven bone	patchy hypomineralized bone		↑ apoptosis; micropetrosis within lacunae
*Fam20c* inactivation in bone, tendon, and skin (193).	6-week-old; male and female	immature and poorly differentiated	↓ processes		↓ type II and type X collagen in growth plate	unmineralized periosteocytic regions, ↓mineral deposition rate	Cortical thinning	
*EphrinB2* deletion in osteocytes (194)	12 week old; female	↑ lacunar density	↔ processes			↑mineralization		
**Osteocyte dendrite changes**								
Sp7 (Osterix) deletion in osteoblasts and osteocytes (195).	8-week-old; male and female	↔ lacunar density		↓ dendrites and connectivity	↑ woven bone	↓ mineralization	↑ cortical porosity	↑ empty lacunae
Bone morphogenetic protein 1 (BMP1) and mammalian tolloid-like 1 (mTLL1) proteinase deletion (196).	17-week-old; male and female	rounder, larger lacunae		↓ number of dendrites	↓ procollagen C-propeptide cleavage	heterogenous mineralization, seams of unmineralized osteoid; ↓tissue mineral density	↓ bone volume, ↑ cortical porosity	
Global CXXC finger protein 5 deficiency (197).	11-week-old; male	↑ osteocyte numbers		↑ dendrite length	↑ woven bone	↑ mineral apposition rate	↑ cortical thickness	
Global deletion of Klotho (FGF23 co-receptor) (183).	6-week-old; sex not reported			↓ number of dendrites				↑ apoptosis
DMP1 and Klotho deficient (183).	6-week-old; sex not reported	↑ osteocyte size, random organization		↓ number of dendrites		↓osteoid area		↑ apoptosis
Sirt3 deletion in osteocytes (182).	3-month-old; male	↓osteocyte density and number		↓ length and number of dendrites per cell			↔ cortical thickness	

Human osteogenesis imperfecta and related mouse models indicate that, in both species, collagen deposition controls how the osteocyte lacuno-canalicular network is established. Quantitative backscattered electron imaging of transiliac cortical bone biopsies from 19 children with hypermineralized bone due to osteogenesis imperfecta type I showed 50% greater osteocyte lacunar density than healthy controls (203). A similar observation was made in osteogenesis imperfecta type V (204), which also exhibits hypermineralization, but due to defects in the *IFITM5* gene. A mouse model with an introduced *Ifitm5* mutation has the same defect in mineralization, along with high osteocyte lacunar density and decreased canalicular density (71), showing conservation of this phenotype between the species.

Mutations in bone morphogenetic protein 1 (*BMP1*) have also been identified as a rare cause of recessive osteogenesis imperfecta (205). Although very few patients with BMP1-associated osteogenesis imperfecta have been identified, in a biopsy study of one patient, thick osteoid seams were present, with hypermineralization in the mineralized bone (206). This heterogeneity of poorly mineralized osteoid and highly mineralized bone was also seen in mice with BMP1 ablation (196) and this was associated with fewer and shorter osteocyte network processes. Fewer and shorter osteocyte processes have also been shown in bone biopsies from patients homozygous for another rare osteogenesis imperfecta-related mutation (*SP7^R316^)* (195). When *Sp7* gene deletion was targeted to the osteoblast lineage in mice, impaired dendrite formation and inter-osteocyte connectivity was also observed (195). In contrast to the IFITM5- and BMP1-deficient mice, the SP7-deficient mice exhibited less mineralization, more woven bone content, and greater cortical porosity than normal (195). This suggests that the defective osteocyte network in SP7 deficiency, whether in murine or human bone, may relate to the greater presence of woven bone, and possibly the retention of an immature cortical bone phenotype. This is like the delayed cortical development in osteocyte-targeted SOCS3-deficient mice described above, which also exhibited low mineralization, more woven bone formation, and an immature, less connected osteocyte network (101, 102).

Mineralization is integral to dendritic process extension as osteocytes mature and become more polarized toward the mineralization front (201). Most studies reporting low osteocyte process number and/or length also observe low mineralization levels (183, 195) or heterogenous mineral surrounding osteocytes (196) (see Table 2). The osteocyte network is also less extensive in mice with delayed mineralization (rickets) (25, 181, 185, 188), disorganized collagen, reduced collagen content (186), or lack of collagen fibril degradation (189). For example, mice with global deletion of the pro-mineralization enzyme dentin matrix protein 1 (DMP1) (183, 184) exhibit incomplete mineralization with thick osteoid seams; the reported reduction in canaliculi likely reflects osteocytes in this unmineralized bone being stopped at the osteoid-osteocyte stage. The *Phex* (phosphate regulating endopeptidase X-linked) knockout mouse model of hypophosphatemic rickets has a similar phenotype (185). While likely to also exist in children with hypophosphatemic rickets due to *PHEX* mutations (207), this has not been reported, probably due to limited access to samples; here mouse models provide useful insights.

Collagen cleavage may also promote osteocyte viability and network formation in lamellar bone. The osteocyte network is less connected, with more empty lacunae and more apoptotic cells, in mice lacking lamellar collagen organization (190) or with impaired collagen α1 and α2 cleavage (191). Osteocytes are also poorly differentiated and retain an immature morphology, with random orientation and shorter dendritic processes, when there is more cross-linking of collagen fibrils (192), when the bone matrix lacks lamellar structure due to shortened collagen fibril length (149), or when type II and X collagen in the growth plate are lacking (193). Whether such collagen defects in human subjects also influence the osteocyte network is unknown.

## Causes of High Cortical Porosity in the Human and Murine Skeleton

There are clinical conditions, aside from age-related and postmenopausal osteoporosis, in which cortical porosity has been reported to increase in humans, and for which there are murine models. These include chronic kidney disease (CKD), primary hyperparathyroidism, some forms of type 2 diabetes, and Gorham-Stout disease. These are complex clinical conditions, with the increase in cortical porosity being but a part of their pathology. We refer the reader to a range of excellent reviews discussing their pathogeneses in detail, including changes to the trabecular network (90, 208-210). Here, we explore whether rodent models of these conditions exhibit changes that reflect the human cortical changes. We note that, where increased porosity has been reported in the rodent models, this is often associated with greater RANKL-induced osteoclast formation within the mature cortex; in some cases, this is due to elevated serum PTH levels (Table 1).

Gorham-Stout disease (GSD, also known as vanishing bone syndrome) is characterized by loss of bone (typically the clavicle, mandible, maxilla, ribs, pelvis) and its replacement by fibrous tissue. This phenomenon is accompanied by inappropriate localized emergence within bone tissue of lymphatic vessels (90) which are usually restricted, in bone, to the periosteum. Two mouse models of GSD have been developed. In one, lymphatic vessel formation was increased by overexpressing the pro-lymphatic VEGF family member (VEGF-C) in the osteoblast lineage in the adult skeleton. This led to “moth-eaten” bones due to increased osteoclast-mediated resorption within the cortex, with increased cortical porosity, analogous to GSD (89). More recently, a mouse model with an activating *KRAS* (Kirsten rat sarcoma viral oncogene homolog) mutation, designed to replicate a mutation identified in a patient with GSD, also exhibited lymphatic vessel development within the bone marrow space, but whether bone loss occurred is not known (211).

Chronic kidney disease (CKD) also exhibits high cortical porosity, as part of a spectrum of skeletal complications (212, 213) likely due to secondary hyperparathyroidism in response to CKD-induced hypocalcemia and hyperphosphatemia (214). Mouse models of CKD, whether induced by 0.2% dietary adenine treatment (84, 86) or the combination of partial renal ablation with high dietary phosphate (85), also exhibit high cortical porosity. In all studies which induced CKD-like symptoms in mice (Table 1), serum PTH levels were significantly higher than baseline. This secondary hyperparathyroidism, while in some cases very mild, might be responsible for inducing abnormal levels of osteoclast formation within intracortical pores resulting in high cortical porosity (84). Both human and mouse models of CKD exhibited a significant rise in serum PTH levels, suggesting a common mechanism inducing increased cortical porosity, even in the absence of osteonal bone.

In primary hyperparathyroidism (PHPT), parathyroid lesions result in continuous PTH secretion (215). Clinical manifestations include a marked elevation of serum calcium, bone pain, and pathological fractures from increased bone resorption (216). A study using HR-pQCT to compare bone microarchitecture of 43 patients with untreated PHPT with healthy age-matched controls discovered greater cortical porosity in PHPT patients (107). In mice, continuous PTH infusion (108) or constitutive activation of PTH receptor (PTH1R) in osteoblast lineage cells (106) also increased cortical porosity, with both characterized by increased osteoclast number and activity. Histological characterization of intracortical pores in mice with osteoblast lineage PTH1R constitutive activation found that these pores contained blood vessels lined with osteoclasts (106). This suggests increased cortical porosity in both murine and human hyperparathyroidism occurs due to increased osteoclast-mediated bone resorption and increased intracortical vasculature; the studies in VEGF-overexpressing mice (above) suggest that the increased vasculature may drive this phenotype.

Elevated PTH has also been suggested as a cause of high cortical porosity in mice with streptozotocin-induced hyperglycemia (110), a phenotype also observed in mice on a high-fat diet (112). In a study of femoral bone from 16 donors diagnosed with type 2 diabetes mellitus (T2DM) and 11 age-matched healthy individuals, half of the T2DM patients exhibited high cortical porosity. By Fourier-transform infrared spectroscopy, the T2DM subgroup with high cortical porosity displayed higher regional mineralization heterogeneity and lower mineral maturity. In contrast, a higher regional mineral-to-matrix ratio was observed in the T2DM subgroup (111). This suggests cortical porosity and bone fragility are not clinical manifestations of all T2DM patients; more investigation is required to understand why cortical porosity increases in some patients with T2DM disease progression, and why they exhibit a change in bone mineralization. Appropriate mouse models could help resolve this.

Regardless of cause, continuously high levels of PTH have a catabolic effect on both cortical and trabecular compartments. This mechanism has been investigated in rats by PTH infusion, which caused a sustained increase in RANKL mRNA and a sustained reduction in OPG mRNA in bone (217). RANKL acts as both as a ligand for RANK and as a signaling receptor that transmits negative and positive feedback signals into RANKL-expressing cells (218). Increased binding of RANKL to RANK on the surface of hematopoietic osteoclast precursors promotes osteoclast maturation and survival (219). OPG is a soluble decoy receptor, which limits osteoclastogenesis by preventing RANKL access to RANK (23). Later cell culture studies showed this was mediated within the osteoblast lineage (220), which expresses the receptor (PTH1R) used by both PTH and its related protein (PTHrP) (221). If OPG levels are reduced, this can also lead to greater cortical porosity, as noted in mice lacking *Wnt16*, either due to global deletion or with deletion targeted to the osteoblast lineage (92).

Besides the membrane-bound form of RANKL expressed on osteoblasts and stromal cells, RANKL can also be supplied pharmacologically as a soluble receptor to stimulate bone resorption (222). This approach also increases cortical porosity as shown by twice-daily recombinant RANKL injections for 10 days in mice (98). Since RANKL was administered after the cortex was consolidated in this study, this suggests intracortical remodeling can be activated in mice, just as it is in humans experiencing either PHPT or secondary hyperparathyroidism due to CKD.

When intracortical resorption is high, as in VEGF-overexpressing mice (96), osteoclast precursors are supplied by the vasculature, leading to more intracortical resorption. Indeed, an unresolved question remains as to whether high cortical porosity originates in increased osteoclastogenesis or in increased vascularization and subsequent provision of osteoclast progenitors. This could be a mechanism common to multiple mouse models with high cortical porosity, including osteoblast/osteocyte-targeted SOCS3-deficient mice, where vascularization and VEGF mRNA levels are high, and even higher in the very porous SOCS3-deficient mouse with G-CSFR deficiency (104). Similarly, in mice with high cortical porosity due to PTH1R constitutive activation in the osteoblast lineage, cortical vascularization was increased (106), and PTH1R signaling stimulation induces vasodilation via VEGF (223). The high cortical porosity of mice with global OPG deletion was also characterized by a greater number of cortical blood vessels (25). Resolving this question could provide new insights into the development of age-related cortical porosity.

In many mouse models with high cortical thickness and high porosity (eg, the C3H mouse strain (78) and the sclerostin knockout mouse (79)), rather than the formation of small lamellar osteons, what is frequently reported is a greater amount of woven bone in the cortex (eg, in the diaphysis). This occurs from woven bone deposition, indicated by fluorescent calcein labeling (78, 93, 100, 104, 115, 121) (see Table 1) rather than by consolidation of a thin cortex. In these models, cortical pores form from resorption of mineralized lamellar bone, and compensatory woven bone deposition provides strength to replace the lost cortical bone. This is characteristic of rapid, coupled bone remodeling, akin to fracture healing, where woven bone formation rates are increased to match the high level of bone resorption, perhaps to compensate for the cortical bone lost during cortical pore enlargement.

## Aging of Trabecular and Cortical Bone in Rodent and Human Bone

### Loss of Human and Murine Trabecular Structure With Aging

With aging, in both men and women, trabecular bone mass declines (36, 224), with greater loss in women than men (225). At a microstructural level, this occurs primarily by loss of trabecular elements, thereby increasing spacing and reducing trabecular interconnectedness (36, 224). Men also exhibit a reduction in trabecular thickness with age (226). Histomorphometric analysis of trabecular bone, again at the iliac crest, showed age-related trabecular bone loss due to a decline in bone formation (225, 227). Recent studies using HR-pQCT at the distal radius suggest the sex-specific microstructural observations may be generalizable to other skeletal sites (228), although neither site is load-bearing. In both male and female C57BL/6 mice, at the distal femur and lumbar vertebrae, trabecular bone loss declines steadily from its peak at 2 months of age until at least 20 months of age; at the distal femur in females, it plateaus at 12 months of age, where trabecular bone has reached near zero (46, 81, 229).

### Cortical Thinning and Increased Cortical Porosity With Aging in Mice and Humans

With aging in humans, cortical bone in both the femoral diaphysis and femoral neck becomes thinner and more porous, with the increased porosity contributing to the thinning (230-232). In rodents, an age-related increase in porosity was first documented in rats in the 1980s, when the appearance of large cortical pores and scalloped endocortical edges, consistent with increased resorption, were noted in femoral diaphyses of 24-month-old male Wistar rats (233). Mice also demonstrate cortical thinning and increased intracortical porosity with aging. To our knowledge, this was first described in the early 2000s in 26-month-old male C57BL/6 mice (81, 229) when both bone formation and resorption were noted within cortical pores (80). Later studies quantified this; by approximately 20 months of age, the increase in cortical porosity is more prominent in female than male mice (82), occurring in the midshaft, metaphysis (82) and femoral neck (160). When the response of cortical bone to aging is studied in mouse and rat models, the animals must be aged to 2 years. This presents a cost challenge, both due to the time required to age the mice, and the need to age a larger cohort, since this is close to the median lifespan of laboratory mice (29 months of age) (234). Mouse models of early aging (eg, SAMP6 mice), may provide a cost-effective model, but whether they reflect normal skeletal aging has been questioned since they do not achieve normal bone mass prior to bone loss (47).

Does the increase in cortical porosity in the aging mouse occur by the same mechanism as in aging human bone? In human bone, microradiography and micro-CT (87, 235) show that cortical pores expand by an increase in pore size, rather than in number (236-238). This suggests intracortical resorption within pre-existing Haversian canals and/or a reduction in intracortical bone formation are the main contributors to increased cortical porosity. In older women, greater cortical porosity is associated with an increase in Haversian canal diameter due to a decrease in bone formation, indicated by lower osteonal wall thickness with no change in osteonal diameter (75). In biopsies from older men, their greater cortical porosity was instead associated with larger osteon diameters, indicating more resorption, with no change in wall thickness (bone formation). This sex-specific difference was not observed in the femoral shaft, which exhibited increased osteon diameter, implying increased bone resorption in both sexes (230). This could reflect a difference in remodeling patterns across the skeleton, perhaps reflecting that the femoral shaft is subjected to greater mechanical load than the iliac crest. In addition, the increase in porosity is not uniform around the femoral diaphysis: more loss occurs in the posterior and anterior regions than on the medial and lateral sides, indicating that mechanical forces may be protective (88, 230). A histomorphometric classification system for intracortical pores in the iliac crest and the fibula (both non-loaded sites), showed increased cortical porosity is largely due to increased remodeling upon existing canals rather than formation of new canals, along with delayed or absent initiation of bone formation (239, 240).

Whether murine cortical porosity results from an increase in pore size rather than number is challenging to resolve since intracortical pores in young mice are typically below the resolution capability of micro-CT, the standard method for screening murine models for skeletal phenotypes. Whether the murine increase in porosity occurs by expansion of endocortical resorption to form new transcortical channels, or by expanding the existing transcortical channels (or both) is also unknown.

### The Osteocyte Network Degenerates With Aging in Mouse and Human

With aging in both human and murine bone, there are reductions in osteocyte number, cell body size, and in the osteocyte dendritic network connectedness.

In human femoral head specimens, the number of viable osteocytes declined significantly with age, with the proportion of viable cells and lacunar density decreasing between younger and older adults (241, 242). When specimens from femoral midshaft, radius, and rib from donors aged 49 to 100 years were assessed, osteocyte lacunar density declined with age only in the femur (243). Osteocyte lacunar density also appeared to decline more significantly with age on the endosteal compared to the periosteal region in both male and female donors (244). There are also contrasting reports finding no association between aging and osteocyte density. For example, there was no effect of age on lacunar density in a high-resolution (synchrotron radiation micro-CT) study of anterior femoral midshafts from female donors aged 20 to 86 years, but osteocyte lacunae in the younger women were flatter, and with a 30% larger volume, than those of the older women (245). This suggests changes in the osteocyte network with age, when they occur, may be site-specific.

The reduction in osteocyte connectivity with aging in human bone does not relate only to a reduction in osteocyte number; the dendritic process number per cell also declined by up to 50% in aged bone compared to younger samples (82). While methods (classic histomorphometry to high-resolution synchrotron scans), and the bones and regions measured vary between these studies, a clear reduction in osteocyte dendritic processes characterizes aging in human bone.

In murine bone, 3 independent studies have reported a less extensive and less connected osteocyte network with aging (82, 181, 182). Aged murine bone had fewer osteocytes and lower osteocyte density per unit of bone (181, 182). The osteocytes present had smaller cell bodies, with a compensatory increase in fluid space within the lacunae (82). As in human bone, osteocyte cell bodies in aged (24-month-old) mice were more rounded than the elliptical osteocytes of 12-month-old mice (182). The network was also less complex, with fewer and shorter osteocytic processes (181, 182) and canaliculi (82). Although fewer, the canaliculi had increased tortuosity with aging, suggesting compromised fluid flow within the network (181).

Osteocyte network degeneration with aging is uneven; some osteocytes in aged mice were found in “islands” with few or no connections to the surrounding osteocytes while other regions retained connectedness, suggesting some regions are more susceptible to degeneration. This reduction in connectivity in both human and murine bone is likely to change cell-to-cell signaling and fluid flow, leading to a changed sensitivity within the network. How the network degrades remains unresolved: does the bone deposited by older individuals incorporate less osteocytes with a more rounded morphology? Is this a response to altered collagen deposition? Is the pre-existing osteocyte network filled by gradual mineralization with aging? Do osteocytes actively maintain their network through their continuing dendritic movement and/or release of bone-degrading enzymes? Does this maintenance process decline with age, allowing the canals to fill with bone material? Whether there are detectable canalicular network remnants in aged bone is unknown.

### Conserved Matrix Maturation of Murine and Human Lamellar Bone

Bone strength is dictated both by the amount of bone present and by its matrix composition, particularly the balance between the 2 major components: collagenous matrix and mineral (bioapatite) crystals. In all species that form bone, during bone formation, osteoid is deposited by osteoblasts, and becomes gradually mineralized over time under the control of a range of noncollagenous proteins produced by osteoblasts and osteocytes (246). The initial (primary) phase of mineralization is rapid: mineral accumulates to reach approximately 70% of maximal levels within a few days (247). Primary mineralization is followed by a slower, secondary phase of mineralization that continues until a maximal mineralization level is reached, or until the bone is removed by resorption, typically during subsequent remodeling.

This progressive mineralization is reflected in an increase in mineral-to-matrix ratio with increasing bone maturity in osteonal bone of humans (248), baboons (249), and intact (247) and ovariectomized (250) rabbits. Although rat and mouse bone rarely exhibit lamellar osteons, they also exhibit progressive mineralization. For example, an increase in mineral-to-matrix ratio is observed in primary lamellar bone in regions undergoing modeling-based bone formation (ie, with no recent prior resorption) in the rabbit (endocortical bone) (250), or mouse (periosteal bone) (194, 251, 252). The same may be true in growing human primary lamellar bone but has not been reported to date. The consistent increase in mineral-to-matrix ratio across the osteon and into murine cortical bone is consistent with murine cortex being described as a *super osteon* (see above, “Haversian vs Non-Haversian Bone: A Function of Life History”).

Bone undergoes multiple maturation processes after osteoid deposition. As mineral-to-matrix ratio increases, there is also an increase in carbonate content, an increase in crystallinity, and compression of the collagen triple helix. Similar changes with distance from the center of the osteon have been observed in multiple species with osteonal bone (including rabbit and baboon) and in maturing primary lamellar bone of both mouse and rabbit (250, 251, 253). Collagen compression has not yet been studied in human bone.

Osteonal bone comprises osteons formed at different timepoints over the course of an individual's life due to remodeling (239, 240). Therefore, any region of osteonal bone contains tissue at different stages of mineralization, with variations in composition dependent on tissue age. Even with this variation, mineral-to-matrix ratio in both cortical and trabecular bone is greater in older humans (254, 255) and baboons (249, 253) than in their younger counterparts. In contrast, bone specimens from postmenopausal women exhibit a lower mineral-to-matrix ratio than premenopausal women (256-258). This may reflect the increase in bone remodeling rate during the menopause, since the greater volume of newly deposited bone would have less time to reach a fully mineralized state before it is resorbed and replaced with a new BMU (259). Mice may be used to resolve these questions, given the consistency in mineral accumulation in murine cortical and osteonal bone, and the ability to provide multiple calcein labels to mice.

As the collagen matrix matures, cross-links form between collagen triple helices. Two types of cross-links exist: enzymatic and nonenzymatic (2). Enzymatic cross-links (ECLs), formed by lysyl hydroxylase and lysyl oxidase, mature into irreducible amino acid–specific cross-links between collagen fibrils (3). These provide additional strength to the bone matrix: human trabecular samples with greater ECL content exhibit greater toughness (4). The ratio between the resulting cross-links can be mapped in situ by Fourier-transform infrared spectroscopy. Although not yet reported in human osteonal bone, this ratio has been reported to increase with matrix maturity in baboon osteonal (5) and mouse cortical bone (6). Consistent with this increase during matrix maturation, ECL content also increases during the first decades of life: human femoral diaphyseal specimens exhibited increasing ECL content until approximately 20 years of age, when it stabilized for the rest of life (7). Whether this also occurs in rodent models is not known, as reports are limited to aged animals, (eg, 24-month-old Fischer F344 rats and 20-month-old BALB/c mice (8, 9)).

Another readily studied family of collagen fibril cross-links are the advanced glycation end-products (AGEs). These form by nonenzymatic glycation of proteins. In contrast to ECLs, these nonenzymatic cross-links form at any point along the collagen fibril and continue to accumulate in bone until old age in humans, rats, and mice (2, 8-10). Higher AGE levels are associated with reduced bone toughness, and this has been reported in human, rat, and mouse cortical bone (8, 9, 11, 12). Consistent with this, bone from women with postmenopausal osteoporosis has been shown to exhibit higher levels of AGEs than that of healthy bone (13, 14), and similar observations have been made in rats with bone loss (15) and a mouse model with type 1 diabetes and bone fragility (16). AGE levels do not appear to change with bone matrix maturation, but this is limited to a single study in rat bone (15); whether it is stable in maturing osteonal bone has not yet been reported. Where it has been studied, rat and mouse models have recapitulated the changes with AGEs seen in humans with tissue maturation and aging, but further investigation is required to confirm whether enzymatic cross-link content stabilizes as a similar age to that of humans, and whether human bone exhibits AGE stability with tissue maturation.

## Guidelines for Measuring and Reporting Trabecular and Cortical Bone in Murine Preclinical Models

Murine models have been excellent models for fundamental discoveries in bone biology, for drug discovery and testing, and for modeling monogenic bone disorders. While their cortical bone is too small to form large, lamellar osteonal structures, their cortex undergoes the same processes as larger species during embryogenesis and growth, exhibits intracortical remodeling during aging and in a range of pathologies, and displays similar mechanisms of matrix maturation as osteonal bone. To make the most of these models for clinical research, in addition to following recommendations to increase reproducibility and reduce bias in animal studies such as the ARRIVE guidelines (260), we suggest 3 key considerations for skeletal research. These are (a) selection of appropriate animal models and controls; (b) detailed reporting of cortical bone phenotypes; and (c) accurate reporting of regions analyzed.

### Select an Appropriate Model, Age, Sex, and Anatomical Location

Mouse models are appropriate for modeling many aspects of human bone development and degeneration, including development of cortical bone. Mouse models have successfully identified genetic contributors, cell signals, and interventions in bone development, cortical growth, metaphyseal consolidation, and age-related degeneration, as well as rare bone diseases. Despite the rarity of osteonal systems in murine bone, mice have been a useful model for understanding how high cortical porosity can emerge, although some details of osteonal bone remodeling will require larger animals, with a thicker cortex, such as the rabbit. Further work is needed to better define some mouse models and strains currently in use, particularly for chronic human conditions where interventions may have stage-specific effects, such as diabetes, CKD, and the peri/postmenopausal transition. In the latter case, since ovariectomy does not reflect the gradual changes of a natural menopause, the 4-vinylcyclohexene diepoxide (VCD) model may prove useful (261), although the only study that assessed its effect on bone found no change in cortical bone mass (262).

Early preclinical studies often showed a single time point and sometimes would combine sexes; since these early phenotypes were generally very dramatic, this was adequate. Later studies began to consider that, just as human bone differs with age and sex, so does mouse bone. This means murine phenotypes also vary with age, depending on whether the pathway disrupted contributes to bone development, growth, or maintenance. Murine phenotypes should be studied and reported at multiple ages. This will inform whether the phenotype relates to embryonic bone development, juvenile bone growth, adult bone homeostasis (between 12 weeks and 26 weeks of age), or age-related degeneration (from 20 months of age). A clear rationale should be included for the age selected, particularly when studying a human condition emerging at a specific age, or a biological pathway more relevant for embryonic development rather than in maintaining the mature skeleton (or vice versa).

Since male mice have greater trabecular bone volume (46) with lower trabecular bone remodeling rates (33), and greater cortical thickness and diameter than female mice (46), some phenotypes are also more readily detectable in one sex than the other. For example, trabecular bone loss is more readily detected in male mice because of their greater bone volume at baseline (141), and the low baseline trabecular bone volume in female mice exhibits a more robust increase with anabolic intermittent PTH treatment (263). Male and female mice, like males and females of all mammalian species, have significantly different bone structure and remodeling and both sexes should be studied and reported (see (264)). In all cases, data must be reported separately for each sex.

If studying therapeutic interventions for adult conditions, young, growing mice or rats (ie, before 12 weeks of age) should be avoided (13) because protective effects may be misleading. For example, when studying antiresorptives in young mice, osteoclast inhibition would protect bone mass by preventing resorption of newly forming trabecular bone at the growth plate. This differs from improving the balance between bone formation and resorption in non-targeted remodeling of trabecular bone, which is the dominant process in the adult skeleton. Conversely, increased osteoclastogenesis in young, growing animals resorbs newly emerging trabeculae at the growth plate (265). This is a fundamentally different process than trabecular bone loss due to increased osteoclastogenesis in a mature animal, where trabeculae are lost due to an imbalance in remodeling.

Another challenge of murine models when studying trabecular bone is the very small region of trabecular bone remodeling in murine tibiae and femora. With age-related trabecular bone loss, female C57BL/6J mice over the age of 26 weeks have almost no trabecular bone remaining in their femur (46), while male mice retain approximately 50% of their femoral trabecular bone volume until 20 weeks of age (46). Murine vertebral trabecular bone mass also reduces with age but retains trabecular bone structure at 20 weeks of ∼50% of maximum in females and 70% in males (46). For this reason, when studying trabecular bone remodeling in aged mice, lumbar vertebrae should be assessed.

### Report Detailed Cortical Phenotypes

In preparing this review, we noted significant under-reporting of cortical bone phenotypes, and lack of detail of how the cortex is measured, particularly in reporting cortical porosity and bone width. Guidelines for cortical micro-CT analysis were published in 2010 (266). These are still relevant and should be used; to these we would add a requirement to report bone lengths and bone widths in both the craniocaudal (anteroposterior) and mediolateral directions (see (267, 268) for examples), since cortical bone shape is genetically controlled and influences the ability of bone to resist load (269).

More detail is also required in reporting of cortical phenotypes. Diaphyseal cortical thickness is becoming more frequently reported, but lacks information about the etiology unless periosteal perimeter, cross-sectional area, and marrow area are reported. These can be measured simply by micro-CT and should be reported at the same site. Depending on the question, such as whether an Erlenmeyer flask morphology exists, it is also relevant to report these at the metaphysis (267). Measurements at the femoral neck could also be reported for cortical phenotypes (160). It is also helpful to report whether the cortical bone is fully consolidated, by reporting porosity, and the amount of bone at low, mid, and high bone density within the maturing cortical bone (101, 104, 270), particularly in conditions of low mineralization, such as vitamin D deficiency (271).

### Report Details of Anatomical Regions Analyzed

Given the variation in trabecular and cortical bone structure, collagen content, mineralization, and osteocyte network complexity with increasing distance from the growth plate, regions of analysis for micro-CT and histomorphometry, including of the osteocyte network, must be reported. Since all these parameters vary with distance from the growth plate, bone lengths must be reported. Many phenotypic reports fail to consider whether the imbalance between resorption and formation arose in the primary spongiosa or occurred in a region reflecting adult trabecular remodeling. This issue was discussed at length when the ovariectomized rat was developed (272), leading to established methods to use aged animals and to avoid the primary spongiosa (273), but is rarely acknowledged in current use of mouse models. When trabecular bone is studied in a growing long bone (regardless of species), the newly formed trabeculae within the primary spongiosa exhibit a distinct region-specific pattern of bone formation and resorption with respect to distance from the growth plate (Fig. 2). For example, near the growth plate there are many osteoclasts resorbing mineralized cartilage, while many osteoblasts are adjacent to this in the region where woven bone is formed, then there is another adjacent region where osteoclasts resorb the primary spongiosa. These regions differ in length between anatomical sites and shorten as growth rate slows.

These different activities in the primary and secondary spongiosa lead to region-specific phenotypes in mouse models (137) and in their responses to therapeutic agents, including PTH and bisphosphonates (160, 274). This must be considered when planning experiments and when reporting data. For example, if the study concerns trabecular remodeling, measurements of trabecular bone volume, osteoblast, and osteoclast surface/number and fluorochrome labels must be made in the secondary spongiosa. In young mice (less than 12 weeks of age), these parameters are unlikely to reflect remodeling, since the trabecular structure is still emerging. This must also be considered in studies of intermittent PTH treatment, where the region of new bone formation is often localized immediately adjacent to the growth plate (see, for example, Fig. 2 in (275)); this reflects establishment of new trabeculae, rather than remodeling, and is of limited relevance to teriparatide use in postmenopausal women (although it would be relevant to managing bone health in pediatrics). The length of the region measured, and its location relative to the growth plate, or other relevant anatomical regions, must be reported in all studies for micro-CT and histomorphometric data. Similarly, studies to investigate adult pathologies, such as testing therapies for established postmenopausal osteoporosis, should use mice ovariectomized after they have reached peak bone mass but before they exhibit an aged skeleton (eg, surgery at least at 16 weeks of age, followed by 4 weeks to lose bone mass).

## Conclusion

Murine preclinical models are excellent models for understanding and treating human bone disease, and for improving our understanding of the basic mechanisms of bone cell function. The reporting of skeletal phenotypes has become more diverse over recent decades. While very early studies primarily reported histological images of trabecular bone phenotypes with quantification by histomorphometry, the emergence of 3D imaging techniques, including micro-CT, provides opportunities for more high-throughput phenotyping. We are now able to investigate a significant range of skeletal properties, broadly covering bone shape, bone mass, bone quality and bone strength. Our review identifies that rodent, including rat and murine, models are appropriate to study many aspects of human bone development and degeneration, including of cortical bone. Mouse models have successfully identified genetic contributors, cell signals, and interventions influencing specific processes, like bone development, cortical growth, metaphyseal consolidation, and age-related degeneration, as well as rare bone diseases. Like any model system, there are limitations to their use, but with careful reporting and appropriate experimental design, mouse models will continue to provide great value to skeletal research.

## Notes

Specialized terms (box of definitions):
**Primary spongiosa:** Newly formed trabeculae in the metaphysis, located immediately adjacent to the cartilaginous growth plate, containing cartilage remnants and woven bone.
**Secondary spongiosa:** More mature trabecular bone that is undergoing remodeling.
**Endosteal bone surfaces:** All the bone surfaces within the marrow cavity, including both trabecular and cortical surfaces.
**Endocortical bone surface:** The inner surface of cortical bone facing the marrow cavity.
**Intracortical bone surface:** Bone surfaces within the network of cortical pores.
**Periosteum:** The outer cortical bone surface (facing the musculature) and the cells that line it.
**Woven bone:** Bone with disordered collagen and osteocyte orientation.
**Lamellar bone:** Bone formed in layers of collagen containing flattened osteocytes oriented along the layers.
**Reversal cells:** A mononuclear osteoblast-lineage cell population that resides on the bone surface during remodeling in the period between bone resorption and bone formation.
**Mechanoresponse:** The ability of cells to change gene expression in response to mechanical loading.

## Abbreviations

AGE: advanced glycation end-product
BMP1: bone morphogenetic protein 1
BMU: basic multicellular unit
CT: computed tomography
DMP1: dentin matrix protein 1
ECL: enzymatic cross-link
G-CSFR: granulocyte colony-stimulating factor receptor
GSD: Gorham-Stout disease
HR-pQCT: high-resolution peripheral quantitative computed tomography
IFITM5: interferon induced transmembrane protein 5
IL-: interleukin
LIF: leukemia inhibitory factor
OPG: osteoprotegerin
PHPT: primary hyperparathyroidism
PTH: parathyroid hormone
PTH1R: parathyroid hormone receptor
PTHrP: parathyroid hormone related protein
RANKL: receptor activator of nuclear factor kappa-B ligand
SOCS3: suppressor of cytokine signaling 3
STAT3: signal transducer and activator of transcription 3
T2DM: type 2 diabetes mellitus
VEGF: vascular endothelial growth factor
